# TACE plus lenvatinib and envafolimab for conversion therapy in unresectable HCC: a prospective pilot study

**DOI:** 10.3389/fimmu.2026.1802197

**Published:** 2026-04-22

**Authors:** Yuhao Su, Yuxin Liang, Deyuan Zhong, Yahui Chen, Ming Wang, Qinyan Yang, Hongtao Yan, Xiaolun Huang

**Affiliations:** Liver Transplantation Center and HBP Surgery, Sichuan Clinical Research Center for Cancer, Sichuan Cancer Hospital & Institute, Sichuan Cancer Center, School of Medicine, University of Electronic Science and Technology of China, Chengdu, China

**Keywords:** conversion therapy, envafolimab, hepatocellular carcinoma, lenvatinib, transarterial chemoembolization

## Abstract

**Background:**

Most patients with hepatocellular carcinoma (HCC) are diagnosed at intermediate or advanced stages, when curative resection is not feasible. Conversion therapy aiming to downstage tumors and enable surgical resection has emerged as a potential strategy. We performed an exploratory, single-arm pilot study to assess the efficacy and safety of transarterial chemoembolization (TACE) combined with lenvatinib and the envafolimab in this setting.

**Methods:**

This single-center, open-label, single-arm pilot trial enrolled patients with Barcelona Clinic Liver Cancer stage B or C unresectable HCC between April and September 2024. Patients received conventional TACE combined with oral lenvatinib and subcutaneous envafolimab until surgical conversion, disease progression, unacceptable toxicity, or death. The primary endpoint was the conversion rate to curative-intent resection. Secondary endpoints included objective response rate (ORR), disease control rate (DCR), pathological response, progression-free survival (PFS), overall survival (OS), and safety.

**Results:**

Fifteen patients were enrolled. According to mRECIST criteria, the ORR was 53.3% and the DCR was 86.7%. Nine patients (60.0%) achieved sufficient tumor downstaging to undergo curative-intent surgery, and all achieved R0 resection. Pathological complete or major response was observed in five resected patients (55.6%). After a median follow-up of 16 months, the estimated median PFS was 12.0 months. One patient died during follow-up, yielding a 1-year OS of 100% and an 18-month OS of 93.3%. Treatment-emergent adverse events were consistent with the known profiles of TACE, lenvatinib, and envafolimab; however, gastrointestinal bleeding was observed and represents an important safety concern that warrants careful risk stratification and proactive management.

**Conclusion:**

In this small pilot study, TACE combined with lenvatinib and envafolimab was associated with encouraging antitumor activity and a relatively high conversion-to-resection rate, with an acceptable safety profile. However, the limited sample size and single-arm design preclude definitive causal conclusions, and gastrointestinal bleeding emerged as an important risk. These preliminary findings suggest the need for validation in larger, controlled trials.

**Clinical Trial Registration:**

https://www.chictr.org.cn/showproj.html?proj=222901, identifier ChiCTR2400081945.

## 1 Introduction

Hepatocellular carcinoma (HCC) remains a major global health challenge and is among the leading causes of cancer-related mortality worldwide (1–3). Despite substantial advances in surveillance strategies and therapeutic approaches, a large proportion of patients, particularly in regions with a high prevalence of chronic hepatitis B virus (HBV) infection such as East Asia, continue to be diagnosed at intermediate or advanced stages of disease (3–5). At these stages, potentially curative treatments, including surgical resection or local ablation, are frequently precluded by excessive tumor burden, vascular invasion, or impaired hepatic reserve (5, 6). As a result, long-term survival outcomes for patients with advanced HCC remain unsatisfactory, highlighting the urgent need for therapeutic strategies that can expand eligibility for curative-intent treatment.

Against this background, conversion therapy has emerged as an important therapeutic paradigm for patients with initially unresectable HCC (uHCC) (7–9). This strategy aims to achieve tumor downstaging through multimodal non-surgical approaches, thereby restoring the possibility of curative resection. Accumulating evidence indicates that patients who undergo successful conversion surgery experience significantly improved survival outcomes compared with those receiving non-curative treatments alone, with R0 resection serving as a critical determinant of long-term prognosis (7, 10).

Transcatheter arterial chemoembolization (TACE) is the standard treatment for intermediate-stage HCC and is also widely used for locoregional control in advanced disease (4, 11–13). By selectively embolizing tumor-feeding arteries, TACE induces ischemic necrosis and reduces tumor viability; however, repeated TACE procedures may compromise hepatic reserve and promote hypoxia-driven angiogenesis, largely through upregulation of vascular endothelial growth factor (VEGF), thereby facilitating tumor recurrence and therapeutic resistance (6, 14, 15). These limitations have constrained the long-term efficacy of TACE monotherapy and prompted the development of combination strategies incorporating systemic treatments. In this context, anti-angiogenic tyrosine kinase inhibitors (TKIs) targeting the VEGF pathway provide a rational complement to TACE. Although the addition of sorafenib to TACE has been shown to improve progression-free survival without consistently extending overall survival (16, 17), lenvatinib, a multi-target TKI with potent anti-VEGF activity, has demonstrated favorable antitumor efficacy and synergistic effects when combined with TACE in real-world and retrospective studies (18, 19). More recently, immune checkpoint inhibitors (ICIs) have transformed the systemic treatment paradigm for HCC (20). TACE-induced tumor necrosis facilitates tumor antigen release and immune cell infiltration, creating a more immunogenic tumor microenvironment that may enhance responsiveness to ICIs (21, 22), and emerging evidence suggests that the combination of TACE with immunotherapy can translate into improved clinical outcomes (23, 24).

Building on these advances, triple-combination strategies integrating TACE, anti-angiogenic therapy, and ICIs have shown promising response, conversion, and survival outcomes in unresectable HCC (25–30), highlighting their potential as an effective conversion approach in selected patients.

Envafolimab (KN035) is a novel single-domain anti-PD-L1 antibody administered via subcutaneous injection and represents the first immune checkpoint inhibitor developed for this route of delivery (31). Early-phase clinical studies have demonstrated that envafolimab exhibits encouraging antitumor activity with an acceptable safety profile across multiple solid tumor types (32–34). In addition to its clinical efficacy, the subcutaneous formulation offers practical advantages, including simplified administration and improved patient convenience, which may be especially advantageous in combination regimens requiring repeated treatment cycles (35).

Based on these considerations, we designed a prospective phase II study to evaluate the efficacy and safety of a triple-combination regimen consisting of envafolimab, lenvatinib, and TACE as conversion therapy for patients with unresectable HCC. The primary objective was to determine whether this multimodal strategy could achieve sufficient tumor downstaging to enable curative resection while maintaining an acceptable safety profile.

## 2 Materials and methods

### 2.1 Study design and patient cohort

This study was a single-center, open-label, single-arm pilot trial conducted at Sichuan Cancer Hospital. Patient enrollment was performed between April 1 and September 30, 2024. Eligible patients were aged 18–70 years with HCC confirmed by imaging or histology according to the American Association for the Study of Liver Diseases (AASLD) criteria. All patients were classified as having intermediate-stage (B) or advanced-stage (C) disease based on the Barcelona Clinic Liver Cancer (BCLC) staging system and were deemed unresectable by a multidisciplinary tumor board owing to tumor extent, anatomical location, vascular involvement, or insufficient future liver remnant.

Key inclusion criteria comprised: (1) at least one measurable lesion according to RECIST version 1.1; (2) have not received any anti-tumor systemic therapy, including but not limited to immunotherapy, targeted therapy, chemotherapy, etc; (3) an Eastern Cooperative Oncology Group (ECOG) performance status of 0 or 1; (4) preserved hepatic function defined as Child–Pugh class A or a score ≤ 7; and (5) adequate hematologic and organ function with an estimated life expectancy of at least 3 months. Key exclusion criteria included: (1) concomitant other malignancies; (2) tumor involvement exceeding 70% of total liver volume; (3) main portal vein trunk thrombosis (Vp4); (4) presence of inferior vena cava or intracardiac tumor thrombus; (5) significant immune deficiency; and (6) symptomatic central nervous system metastases. According to the research protocol, adequate organ function was defined as: (1) Blood routine: WBC≥3.0×10^9/L; ANC≥1.5×10^9/L; PLT≥100×10^9/L; HGB≥90 g/L; (2) Liver function: AST ≤ 2.5×ULN; ALT ≤ 2.5×ULN; TBIL ≤ 1.5×ULN; (3) Renal function: Cr ≤ 1.5×ULN or CrCl ≥60 mL/min; (4) Coagulation function: INR ≤ 1.5, APTT ≤ 1.5×ULN; (5) No obvious abnormality in electrocardiogram.

All participants provided written informed consent prior to enrollment. The study protocol was approved by the institutional ethics committee (Approved No. of ethic committee:S20210046) and conducted in accordance with the Declaration of Helsinki and Good Clinical Practice guidelines. The trial was registered at http://www.chictr.org.cn (ChiCTR2400081945).

### 2.2 Definition of initially unresectable HCC

Initially unresectable HCC was defined as disease deemed unsuitable for immediate curative-intent hepatectomy at baseline following multidisciplinary team (MDT) evaluation. Unresectability was determined on the basis of either surgical or oncologic considerations. Surgical unresectability included inadequate hepatic functional reserve, clinically significant portal hypertension, insufficient future liver remnant, poor performance status, or major comorbidities precluding safe liver resection. Oncologic unresectability included tumor burden or distribution not amenable to upfront curative resection, including multifocal bilobar disease, major macrovascular invasion, extrahepatic metastasis, or tumor extent for which R0 resection was not technically feasible or was unlikely to provide meaningful oncologic benefit. All patients were assessed by an MDT consisting of hepatobiliary surgeons, interventional radiologists, hepatologists, medical oncologists, and radiologists, and the final determination of unresectability was based on consensus clinical judgment integrating liver function, remnant liver volume, tumor anatomy, and disease biology.

### 2.3 Treatment protocol

All enrolled patients received a triple-combination regimen consisting of TACE, lenvatinib, and envafolimab. Epirubicin was used as the principal chemotherapeutic agent (Brand Name: Pharmorubicin^®^, Manufacturer: Pfizer Inc., Country of Origin: China). The dose was individualized according to tumor burden, liver function, and treatment extent, usually controlled within the range of international consensus and regional practice recommendations. In short, the selective arterial catheter was advanced into the tumor supply artery, and then gelatin sponge embolic agent was injected into the artery for embolization until a stasis state was reached. TACE procedures were repeated on demand at approximately 6-week intervals, based on radiologic tumor response and investigator assessment, such as the presence of residual viable tumor exceeding 50% or the emergence of new lesions.

Envafolimab (Brand Name: Enweida^®^, Manufacturer: Sichuan Siliukangrui Pharmaceutical Co., Ltd., Country of Origin: China) was administered subcutaneously at a fixed dose of 300 mg once every 3 weeks, initiated within one week after the first TACE procedure. Lenvatinib (Brand Name: Lenvima^®^, Manufacturer: Eisai, Country of Origin: Japan) was administered orally once daily at a dose of 8 mg for patients weighing < 60 kg and 12 mg for those weighing ≥ 60 kg, starting on day 1 of systemic therapy.

Patients continued combination treatment until one of the following criteria was met: achievement of surgical resectability and subsequent curative-intent resection, radiologic disease progression, unacceptable toxicity, withdrawal of consent, or death. For patients who achieved resectability, hepatectomy was performed as soon as clinically feasible, typically 4–6 weeks after the last TACE session to allow adequate recovery. No adjuvant envafolimab or lenvatinib was administered postoperatively according to the study protocol, and patients entered follow-up for recurrence surveillance.

Tumor resectability was evaluated by a multidisciplinary team based on radiologic response and clinical status. Surgical eligibility was defined by the following criteria: (1) preserved liver function and general condition, indicated by a Child–Pugh score ≤ 7, indocyanine green retention rate at 15 minutes (ICG R15) ≤ 10%, and ECOG performance status of 0–1; (2) complete response (CR) and partial response (PR), or stable disease (SD) maintained for at least 2 months, provided all other resectability criteria are met according to modified RECIST (mRECIST); (3) sufficient future liver remnant volume, defined as ≥ 35% of standard liver volume in patients without cirrhosis and ≥ 45% in those with cirrhosis; (4) regression or inactivation of vascular tumor thrombi with technical feasibility for curative resection and an intent to achieve R0 margins; and (5) absence of other contraindications to surgery. The detailed MDT decision template is provided in **Supplementary Table 1**. Hepatectomy was performed in medically fit patients in accordance with standard surgical practice. Patients who did not undergo resection continued treatment until protocol-defined endpoints. Patients with active viral hepatitis were permitted, and those with hepatitis B virus infection received antiviral therapy.

### 2.4 Endpoints and assessments

The primary endpoint was the conversion rate to curative surgery, defined as the proportion of patients who successfully underwent R0 resection following combination therapy. Secondary endpoints included objective response rate (ORR), disease control rate (DCR), R0 resection rate, pathological response, progression-free survival (PFS), overall survival (OS), safety, and changes in peripheral immune cell subsets. ORR was defined as the proportion of patients achieving complete response or partial response as the best overall response according to mRECIST criteria. DCR was defined as the proportion achieving complete response, partial response, or stable disease. Pathological response was assessed in resected specimens and categorized as pathological complete response (pCR), defined as the absence of viable tumor cells, or major pathological response (MPR), defined as ≤ 10% residual viable tumor cells. PFS was defined as the time from treatment initiation to radiologic progression, postoperative recurrence, or death from any cause, whichever occurred first. OS was defined as the time from treatment initiation to death from any cause. Safety was evaluated by monitoring treatment-related adverse events, graded according to the Common Terminology Criteria for Adverse Events version 5.0 (36).

Peripheral blood was collected at baseline and at the prespecified post-treatment timepoint defined as 3–4 weeks after the last treatment cycle or within 7 days prior to surgery for patients. Changes in immune cell counts were explored in relation to radiologic response and surgical conversion. Cytotoxic T cells were defined as CD3+CD8+ cells, helper T cells as CD3+CD4+ cells, and NK cells as CD3−CD16+CD56+ cells. Antibody clones/fluorochromes, suppliers, cytometer configuration, gating strategy, and analysis software are reported in **Supplementary Table 2** in accordance with MIFlowCyt recommendations (37).

Radiologic tumor assessment was performed using contrast-enhanced computed tomography or magnetic resonance imaging at 6-week intervals. Tumor response was evaluated according to mRECIST criteria by an independent radiologist (38). Resected specimens were examined by experienced hepatopathologists to assess tumor viability, necrosis, microvascular invasion, and histologic differentiation.

### 2.5 Statistical analysis

This exploratory pilot trial employed a single-arm design with the objective of assessing whether the conversion rate exceeded historical benchmarks of approximately 20%. Given the exploratory nature of the study, no formal sample size calculation was performed. A total of 15 patients was considered sufficient to provide preliminary efficacy and safety signals. Efficacy endpoints, including ORR and conversion rate, were reported with exact 95% confidence intervals calculated using the Clopper–Pearson method. Time-to-event outcomes were estimated using the Kaplan–Meier method. For within-subject pre–post immune cell count comparisons, we used a Wilcoxon signed-rank test (two-sided) given the small sample size; between-group comparisons of change values (Δ) were assessed using the Mann–Whitney U test. Receiver operating characteristic (ROC) curve analysis was performed to evaluate the predictive performance of immune cell changes for surgical conversion. AUCs and 95% CIs were calculated using the DeLong method (or bootstrap, as specified). Statistical analyses were conducted using R software (version 4.2) and SPSS (version 26).

## 3 Results

### 3.1 Patient characteristics

**Figure 1:**
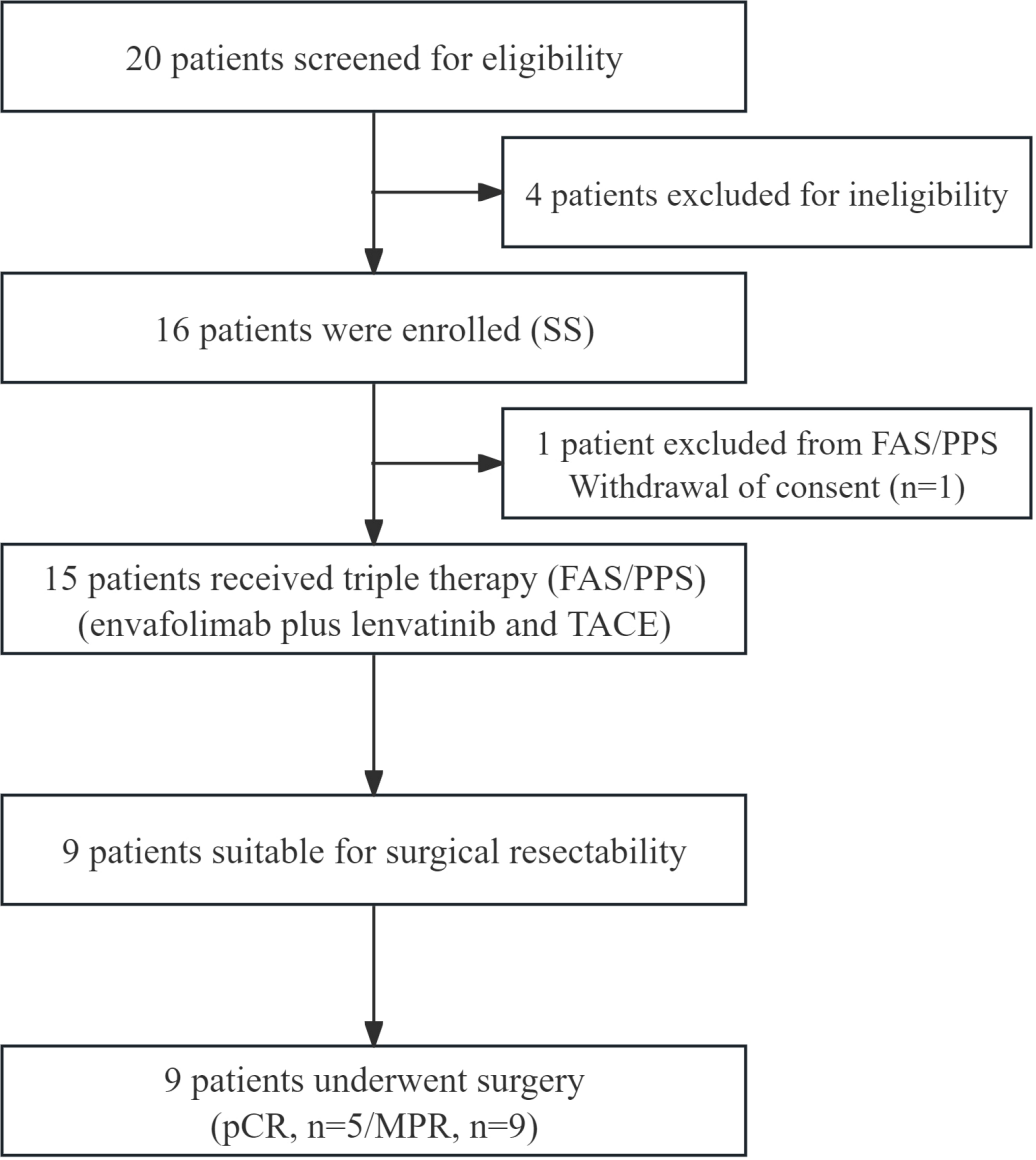
Flowchart of patient enrollment. SS, safety analysis set; FAS, full analysis set; PPS, per protocol set; TACE, transarterial chemoembolization; pCR, pathologic complete response; MPR, major pathological response.

Between April and September 2024, a total of 15 patients with uHCC were enrolled and received at least one cycle of the triple-combination therapy. The patient enrollment process is illustrated in **Figure 1**. Baseline demographic and clinical characteristics are summarized in **Table 1**. The median age was 54 years (range, 33–70 years), and 13 patients (86.7%) were male. Chronic hepatitis B virus infection was the predominant etiologic factor, present in 12 patients (80.0%), and 10 patients (66.7%) had underlying liver cirrhosis. All patients had preserved performance status, with an ECOG score of 0 in 9 patients (60.0%) and 1 in 6 patients (40.0%). Liver function was classified as Child–Pugh class A in 11 patients (73.3%) and class B in 4 patients (26.7%). According to the BCLC staging system, 5 patients (33.3%) had intermediate-stage disease (stage B) and 10 patients (66.7%) had advanced-stage disease (stage C) at baseline. Multifocal tumors were observed in 10 patients (66.7%). The median diameter of the largest tumor lesion was 5.5 cm (range, 2.7–12.0 cm). Portal vein tumor thrombosis at the branch or segmental level (Vp1–Vp3) was present in 10 patients (66.7%), whereas involvement of the main portal vein trunk (Vp4) was absent in all cases by study design. No patient had detectable extrahepatic metastases at enrollment. Baseline serum alpha-fetoprotein levels exceeded 400 ng/mL in 6 patients (40.0%), with a median value of 277.6 ng/mL (range, 3.7–47,531 ng/mL). All patients were naïve to systemic anticancer therapy at study entry.

**Table 1:** Patient baseline characteristics.

Characteristics	All patients (n=15)
Age (years), median (range)	54 (33 to 70)
Sex, n (%)	
Male	13 (86.7%)
Female	2 (13.3%)
BMI, median (range)	23.7 (20.8 to 28.9)
Etiology, n (%)	
HBV positive	12 (80.0%)
HBV negative	3 (20.0%)
Cirrhosis, n (%)	
Present	10 (66.7%)
Absent	5 (33.3%)
ECOG-PS, n (%)	
0	9 (60.0%)
1	6 (40.0%)
BCLC stage, n (%)	
B	5 (33.3%)
C	10 (66.7%)
ALBI Score, median (range)	-2.62 (-2.99 to -1.81)
ALBI grade, n (%)	
1	7 (46.7%)
2	8 (53.3%)
CNLC staging, n (%)	
IIa	2 (13.3%)
IIb	3 (20.0%)
IIIa	8 (53.3%)
IIIb	2 (13.3%)
Child-Pugh class, n (%)	
A	11 (73.3%)
B	4 (26.7%)
Tumor number, n (%)	
Single	5 (33.3%)
Multiple	10 (66.7%)
Largest tumor diameter (cm), median (range)	5.5 (2.7 to 12)
PVTT, n (%)	
Present	10 (66.7%)
Absent	5 (33.3%)
AFP (ng/mL), median (range)	277.6 (3.7 to 47,531)
AFP (ng/mL), n (%)	
≥400	6 (40.0%)
<400	9 (60.0%)

HBV, Hepatitis B Virus; ECOG-PS, Eastern Cooperative Oncology Group performance status; ALBI, albumin-bilirubin; CNLC, China Liver Cancer; BCLC, Barcelona Clinic Liver Cancer; AFP, alpha-fetoprotein; BMI, body mass index; PVTT, portal vein tumor thrombosis.

### 3.2 Tumor response

**Figure 2:**
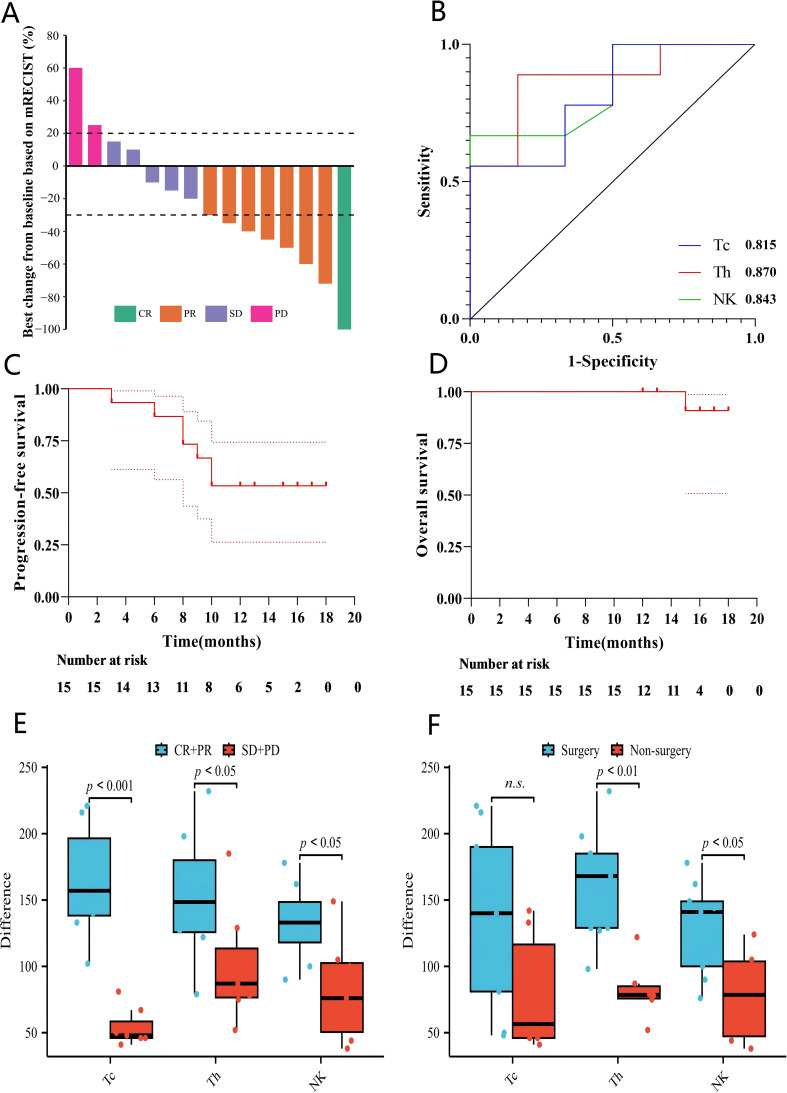
Patient survival, conversion, and immune cell analyses. **(A)** Best tumor response for each patient. Dashed lines indicate the mRECIST thresholds for PR (−30%) and PD (+20%). **(B)** ROC curves showing the predictive performance of changes in three immune cell subsets for surgical conversion. Kaplan–Meier survival curves of progression-free survival **(C)** and overall survival **(D)** for the entire cohort. Box plots of the change values of three types of immune cells, along with the **(E)** efficacy and **(F)** surgical conversion rate. Tc, cytotoxic T cells; Th, helper T cells; NK cells, natural killer cells.

Radiologic tumor responses were assessed according to mRECIST criteria. Among the 15 evaluable patients, 1 patient achieved a complete response and 7 patients achieved a partial response, resulting in an objective response rate of 53.3%. An additional 5 patients experienced stable disease as their best response, yielding a disease control rate of 86.7%. Two patients (13.3%) developed progressive disease during treatment. Tumor responses were generally observed early in the treatment course, frequently at the first scheduled imaging assessment. The maximum percentage change in target lesion size for each patient is illustrated in the waterfall plot shown in **Figure 2A**. Patients achieving partial response exhibited tumor size reductions ranging from approximately 30% to 70%, whereas the patient with complete response demonstrated complete radiologic disappearance and necrosis of target lesions. In several patients classified as having stable disease, substantial tumor necrosis was nonetheless observed, which subsequently facilitated surgical resection. In exploratory subgroup analysis, we observed that 6 of 10 patients with BCLC stage C achieved an objective response, whereas 2 of 5 patients with BCLC stage B met the criteria for ORR.

### 3.3 Conversion to surgery

The primary endpoint of conversion to resectability was achieved in 9 of 15 patients, corresponding to a conversion rate of 60.0%. These patients, who were initially deemed unresectable, demonstrated sufficient tumor regression and stabilization to permit surgical evaluation and were subsequently considered eligible for curative-intent resection by the multidisciplinary team.

The median interval from treatment initiation to the determination of resectability was 3 months, following 1–3 TACE sessions and a median of four cycles of envafolimab. All nine patients proceeded to surgery and underwent hepatectomy with curative intent. Eight patients underwent partial hepatectomy, ranging from segmentectomy to hemihepatectomy according to tumor location, and one patient with multifocal disease underwent extended resection combined with local ablation of small satellite lesions. We have added **Supplementary Table 3** to summarize the clinical details and reasons for MDT records of 6 patients who did not undergo surgical resection, such as future residual liver insufficiency, residual vascular involvement unsuitable for R0 resection, comorbidities, or patient preferences.

All resected patients achieved microscopically margin-negative (R0) resection. Pathological examination revealed extensive treatment-related tumor necrosis in the majority of resected specimens. Pathological complete response, defined as the absence of viable tumor cells, was observed in 2 patients (22.2% of those resected). Major pathological response, defined as ≤ 10% residual viable tumor cells, was observed in 3 patients (33.3%). The remaining resected specimens demonstrated variable degrees of tumor necrosis ranging from approximately 40% to 80%. Despite substantial treatment effect, microvascular invasion was identified in 8 of the 9 resected tumors (88.9%). All resected tumors were classified as moderately or poorly differentiated HCC on histologic examination. In exploratory subgroup analysis, we observed that successful conversion was achieved in 7 of 10 patients with BCLC stage C disease and in 2 of 5 patients with BCLC stage B disease.

### 3.4 Survival outcomes

As of September 30, 2025, the median follow-up duration from treatment initiation was 16 months (range, 12–18 months) among surviving patients. Kaplan–Meier estimates of PFS and OS are shown in **Figures 2C**, **D**, respectively. For the entire cohort, the estimated median PFS was 12.0 months. The PFS rates at 6 and 12 months were 83.3% and 56.7%, respectively, and approximately 56.7% of patients remained free of progression or recurrence at 18 months.

Among the nine patients who underwent surgical resection, six patients (66.7%) remained recurrence-free at last follow-up, with recurrence-free survival ranging from 12 to 17 months. Three patients (33.3%) developed tumor recurrence at a median of 6 months after surgery, with all recurrences confined to the liver. Among the six patients who did not undergo resection, four experienced disease progression at 8, 8, 9, and 10 months, respectively, whereas two patients remained progression-free through 15–18 months of follow-up while continuing therapy.

Only one patient died during the follow-up period but was not considered treatment-related. The patient had a dual risk of cirrhosis and portal hypertension. The patient underwent conversion surgery after achieving stable disease and died at 15 months due to acute gastrointestinal bleeding without evidence of tumor recurrence. The median overall survival was not reached. The estimated 1-year overall survival rate was 100%, and the 18-month overall survival rate was 93.3%.

### 3.5 Safety and tolerability

All 15 patients were included in the safety analysis. Treatment-related adverse events are summarized in **Table 2**. All patients experienced at least one adverse event of any grade. The majority of adverse events were consistent with the known safety profiles of TACE, lenvatinib, and immune checkpoint inhibition, and no unexpected toxicities were observed.

**Table 2:** Treatment-related adverse events (TRAEs).

Adverse event	Any grade, n (%)	Grade ≥3, n (%)
Any TRAE	15 (100%)	8 (53.3%)
Aspartate aminotransferase increased	11 (73.3%)	2 (13.3%)
Alanine aminotransferase increased	10 (66.7%)	2 (13.3%)
White blood cell count decreased	6 (40.0%)	0
Platelet count decreased	5 (33.3%)	1 (6.7%)
Neutrophil count decreased	5 (33.3%)	1 (6.7%)
Hypertension	4 (26.7%)	0
Palmar-plantar erythrodysesthesia syndrome	3 (20.0%)	1 (6.7%)
Hypothyroidism	4 (26.7%)	0
Hyperthyroidism	2 (13.3%)	0
Thyroid stimulating hormone increased	5 (33.3%)	0
Hypoalbuminemia	4 (26.7%)	0
Gastrointestinal bleeding	6 (40.0%)	1 (6.7%)
Rash or dermatitis	2 (13.3%)	0
Fatigue	4 (26.7%)	0
Fever	3 (20.0%)	0

To further characterize toxicity management, most adverse events were handled with supportive care, temporary treatment interruption, or dose modification rather than permanent discontinuation of the entire regimen. Elevations in liver enzymes were the most frequent adverse events, with aspartate aminotransferase and alanine aminotransferase increases occurring in 73.3% and 66.7% of patients, respectively; most events were grade 1–2, while grade 3 elevations were observed in two patients for each parameter, and no grade 4 hepatic abnormalities occurred. Transient post-treatment liver enzyme elevations were managed conservatively with close biochemical monitoring and hepatoprotective/supportive therapy. Hematologic toxicities included decreases in white blood cell and platelet counts in 40.0% and 33.3% of patients, respectively. One patient developed grade 3 neutropenia without infection. One patient with grade 3 thrombocytopenia underwent temporary interruption of lenvatinib, and one patient with grade 3 palmar–plantar erythrodysesthesia syndrome required lenvatinib dose reduction; both events improved after intervention. Immune-related adverse events were predominantly mild, mainly thyroid dysfunction, with no immune-related hepatitis, pneumonitis, or colitis reported. Gastrointestinal bleeding occurred in 6 patients, including 1 grade 3 event. Management was individualized according to severity and clinical context, including temporary withholding of anti-angiogenic therapy, hemodynamic assessment, proton-pump inhibitor and/or hemostatic treatment, and endoscopic evaluation/intervention when clinically indicated. We have verified CTCAE v5.0 grading and updated **Supplementary Table 4** to provide event-level details, including timing relative to each treatment component and surgery, management, and investigator attribution. Given the high proportion of cirrhosis and portal hypertensive risk in this cohort, these bleeding events are discussed in the context of baseline portal hypertension/variceal risk factors and anti-angiogenic therapy exposure. No treatment-related deaths occurred, and no patient permanently discontinued all study treatments due to adverse events.

### 3.6 Immune cell dynamics and association with treatment efficacy

Peripheral immune cell subsets were evaluated before treatment initiation and after completion of the last treatment cycle. Changes in cytotoxic T cells, helper T cells, and natural killer cells were calculated for each patient.

Patients achieving objective response (CR or PR) demonstrated significantly greater increases in cytotoxic T cells, helper T cells, and natural killer cells compared with patients with stable or progressive disease (**Figure 2E**). Specifically, increases in helper T cells and natural killer cells were also significantly greater in patients who successfully underwent surgical conversion compared with those who did not (**Figure 2F**), whereas changes in cytotoxic T cells did not reach statistical significance between surgical and non-surgical groups.

Receiver operating characteristic curve analysis demonstrated that changes in helper T cells, natural killer cells, and cytotoxic T cells exhibited good discriminatory performance for predicting surgical conversion, with areas under the curve of 0.870, 0.843, and 0.815, respectively (**Figure 2B**). Analysis results of immune cell changes (Δ), therapeutic efficacy, and surgical transformation are shown in **Supplementary Table 5**.

## 4 Discussion

In this prospective, single-arm pilot study, we evaluated a triple-combination strategy comprising TACE, lenvatinib, and the subcutaneous PD-L1 inhibitor envafolimab as conversion therapy for patients with uHCC. Our findings indicate that this multimodal regimen can induce meaningful tumor regression and facilitate curative-intent resection in a subset of patients initially deemed unsuitable for surgery. Although follow-up duration remains limited, early survival outcomes appear encouraging. The estimated 18-month overall survival rate exceeded that typically reported in historical cohorts of advanced HCC treated with systemic therapy alone (39). Although durable outcomes were observed in several resected patients, the single-arm design precludes causal attribution of survival benefit to surgery. Notably, some non-resection patients also experienced prolonged disease control on continued therapy, highlighting the potential for selection bias and time-dependent confounding. Comparative studies are needed to determine the incremental benefit of surgery over optimized systemic/locoregional therapy in this setting.

The most clinically significant result was a conversion-to-surgery rate of 60%. Historically, conversion rates following locoregional therapy or systemic therapy alone have generally ranged from approximately 15% to 40% in patients with intermediate or advanced HCC, depending on patient selection, treatment modality, and institutional resectability criteria (7, 40–42). Within this context, the conversion rate observed in our study appears numerically higher than those reported in several prior investigations of dual or triple combination strategies (43–46), although such cross-study comparisons should be interpreted with caution given heterogeneity in study design, baseline tumor burden, and definitions of resectability. Importantly, the universal achievement of R0 resection among converted patients is of particular clinical relevance, as margin-negative surgery remains a prerequisite for durable disease control and potential cure in HCC (47–49).

According to mRECIST criteria, the triple-combination regimen achieved an ORR of 53.3% and a DCR of 86.7%, reflecting a moderate-to-high level of antitumor activity with effective disease stabilization. These outcomes are broadly consistent with those reported in recent prospective studies evaluating similar multimodal approaches. For example, the multicenter study ChiCTR2100050410 reported a higher mRECIST ORR of 76.4% with TACE plus lenvatinib and camrelizumab, while the DCR (85.5%) was comparable to that observed in our cohort (43). Similarly, the phase II ChiCTR2100049829 study reported mRECIST ORR and DCR of 67% and 91% (50), respectively, with TACE plus atezolizumab and bevacizumab. In contrast, IMbrave150, which established atezolizumab plus bevacizumab as standard first-line systemic therapy, reported a lower mRECIST ORR of approximately 35% and a DCR of 73.6% in the overall intention-to-treat population, although outcomes in the BCLC-B subgroup (ORR 50%, DCR 85%) were more closely aligned with our findings (51).

The heterogeneity in radiologic response across these studies likely reflects differences in patient selection, including BCLC stage distribution, tumor burden, and conversion-oriented treatment intent, as well as variations in TACE techniques, systemic drug combinations, and imaging assessment protocols. Notably, despite not achieving the highest ORR among comparable studies, our regimen resulted in a favorable conversion-to-surgery rate, suggesting that radiologic response alone may not fully capture the biological effects most relevant to resectability. Treatment-induced tumor devitalization, regression or inactivation of vascular tumor thrombi, and preservation of hepatic functional reserve may together play a critical role in enabling successful surgical downstaging.

In exploratory immune analyses, dynamic changes in peripheral immune cell subsets were associated with both radiologic response and successful surgical conversion, providing biological insight into the efficacy of the triple-combination regimen. Patients achieving objective response exhibited greater increases in cytotoxic T cells, consistent with the established role of CD8^+^ T cells as primary effectors of immune checkpoint blockade–mediated tumor control in HCC. TACE-induced tumor necrosis may enhance antigen release and presentation (4, 13), while lenvatinib-mediated VEGF inhibition can alleviate hypoxia-driven immunosuppression and improve immune cell infiltration, thereby augmenting the antitumor activity of PD-L1 blockade (18). Notably, in the surgery versus non-surgery comparison, increases in helper T cells and NK cells were significantly greater, whereas cytotoxic T-cell changes showed a non-significant trend (P = 0.101). Therefore, these immune findings should be interpreted as exploratory and hypothesis-generating. Helper T cells are critical for sustaining effective cytotoxic responses and coordinating broader immune remodeling through cytokine signaling, whereas natural killer cells contribute to rapid, antigen-independent tumor cell elimination and may facilitate tumor devitalization rather than size reduction alone (52–54). This coordinated activation of adaptive and innate immunity may help explain why some patients classified as having stable disease by mRECIST nonetheless achieved sufficient biological response to permit curative-intent resection.

The safety profile of the triple-combination regimen was generally consistent with the known toxicities of TACE, lenvatinib, and immune checkpoint inhibition (20, 55, 56). Importantly, liver function was largely preserved throughout treatment, which is a critical consideration in patients with underlying cirrhosis undergoing intensive multimodal therapy. The subcutaneous administration of envafolimab was well tolerated and was not associated with infusion-related reactions, offering a practical advantage that may improve treatment feasibility and patient convenience in real-world settings (57). While the overall toxicity spectrum was broadly consistent with known profiles, the observed gastrointestinal bleeding incidence constitutes a clinically important safety signal. This finding contrasts with data from a prospective study that reported rare serious adverse events of gastrointestinal bleeding and suggested that baseline portal hypertensive risk and event definitions/timing may materially influence observed bleeding rates (44). Future studies should incorporate standardized bleeding-risk assessment and prophylactic strategies in line with portal hypertension guidance. An additional challenge in interpreting safety in this study is that toxicity attribution is inherently complex in a triple-combination regimen. Some adverse events are more plausibly linked to a specific component: transient transaminase elevation, fever, and post-embolization constitutional symptoms are commonly seen after TACE and may reflect ischemic tumor necrosis and non-tumoral liver injury; hypertension, palmar–plantar erythrodysesthesia, proteinuria, and bleeding tendency are more consistent with the anti-angiogenic effects of lenvatinib; and thyroid dysfunction or other inflammatory toxicities are more suggestive of immune-related effects associated with envafolimab. However, in real-world conversion therapy these boundaries are often blurred. For example, liver injury may be amplified by the combination of embolization-related ischemia and systemic therapy, while gastrointestinal bleeding may reflect the interaction of baseline cirrhosis/portal hypertension, variceal vulnerability, TACE-related hepatic decompensation, and VEGF-pathway inhibition. Accordingly, causality in our cohort should be interpreted as investigator-assessed rather than definitive. This attribution difficulty is an important limitation of multimodal safety evaluation and supports the need for prospective studies with predefined toxicity adjudication, standardized bleeding-risk assessment, and protocolized rules for treatment interruption, dose reduction, and re-challenge.

Several limitations of this study warrant careful consideration. First, the single-center, non-randomized design and small sample size limit the generalizability of the findings and preclude formal comparisons with standard-of-care treatments, while also restricting statistical power and definitive causal inference. Given the small sample size, ROC analyses are prone to optimism and overfitting. The wide 95% confidence intervals around AUC estimates indicate substantial uncertainty and limited transportability; external validation in larger cohorts is required before clinical use. Second, surgical decision-making was based on multidisciplinary evaluation within a high-volume center, which may not be directly applicable to institutions with differing surgical expertise or resectability thresholds. Third, follow-up duration remains relatively short, and long-term outcomes, including late recurrence and overall survival, require further observation. At the same time, future research should focus on variables such as PVTT, AFP levels, and BCLC grading, and subgroup analysis can help screen specific beneficiary populations. Finally, the exploratory immune analyses were constrained by the limited sample size and predefined sampling time points, and immune profiling was restricted to peripheral blood, which may not fully capture the spatial and temporal heterogeneity of the intratumoral immune microenvironment. In addition, functional phenotyping of immune cells and tissue-based correlative analyses were not performed. Accordingly, the immunological findings should be interpreted as hypothesis-generating and warrant validation in larger, multicenter cohorts incorporating longitudinal sampling and integrated tumor–immune profiling.

## 5 Conclusion

In conclusion, this prospective study suggests that a triple-combination regimen comprising TACE, lenvatinib, and the subcutaneous PD-L1 inhibitor envafolimab shows potential as a conversion strategy for patients with initially uHCC. However, gastrointestinal bleeding was relatively common, underscoring the need for rigorous monitoring and prophylaxis of portal hypertension–related bleeding in future applications. Although the findings should be interpreted cautiously given the single-arm design, limited sample size, and relatively short follow-up, they provide prospective, hypothesis-generating evidence supporting the integration of locoregional therapy with anti-angiogenic and immune checkpoint inhibition to expand surgical eligibility in carefully selected patients. Larger, controlled trials with longer follow-up and careful patient selection are essential to confirm the safety and benefit of this multimodal strategy and to define its optimal role in advanced HCC management.

## Data Availability

The raw data supporting the conclusions of this article will be made available by the authors, without undue reservation.
