# Supplementary material for: TACE plus lenvatinib and envafolimab for conversion therapy in unresectable HCC: a prospective pilot study
Source: Front Immunol. 2026 Apr 22;17:1802197. doi: 10.3389/fimmu.2026.1802197 (PMC13143888; doi:10.3389/fimmu.2026.1802197)
Supplement: Supplementary file 1 [file Supplementaryfile1.zip › Supplementary Table 2.docx]

**Supplementary Table 2. Flow cytometry assay conditions for absolute quantification of peripheral blood Th, Tc, and NK cells**

| **Item** | **Specification** |
| --- | --- |
| Sample | Peripheral venous blood collected into EDTA tubes and processed as whole blood |
| Timing | Samples were stained within 24 h of collection and acquired within 6 h after staining |
| Antibody panel | CD3 FITC (SK7), CD16/CD56 PE, CD45 PerCP-Cy5.5 (2D1), CD4 PE-Cy7 (SK3), CD19 APC (SJ25C1), and CD8 APC-Cy7 (SK1); all from BD Biosciences |
| Red blood cell lysis | BD FACS Lysing Solution, used according to the manufacturer’s instructions |
| Instruments | BD FACSLyric flow cytometer equipped with 405-, 488-, and 640-nm lasers using BD FACSuite™ Clinical software (Becton, Dickinson and Company, San Jose, CA, USA) |
| Quality control | Daily instrument setup and quality control were performed using manufacturer-recommended setup beads |
| Compensation and controls | Compensation was established using single-stained controls; gating thresholds were confirmed using negative and/or FMO controls where applicable |
| Gating strategy | Lymphocytes were identified on CD45 versus SSC, followed by separation into CD3+ T cells and CD3− lymphocytes |
| Cell subset definitions | Th, CD3+CD4+; Tc, CD3+CD8+; NK, CD3−CD16+CD56+ |
| Absolute counting method | Dual-platform calculation based on flow cytometric percentages combined with absolute lymphocyte counts from routine complete blood counts |
| Reported variables | Absolute counts |
