# Supplementary material for: TACE plus lenvatinib and envafolimab for conversion therapy in unresectable HCC: a prospective pilot study
Source: Front Immunol. 2026 Apr 22;17:1802197. doi: 10.3389/fimmu.2026.1802197 (PMC13143888; doi:10.3389/fimmu.2026.1802197)
Supplement: Supplementary file 1 [file Supplementaryfile1.zip › Supplementary Table 5.docx]

**Supplementary Table 5. Analysis results of immune cell changes (Δ), therapeutic efficacy, and surgical transformation.**

| **Variables** | **CR+PR ( M± S)** | **SD+PD ( M± S)** | **P value** | **Surgery ( M± S)** | **Non-surgery ( M± S)** | **P value** | **AUC (95% CI)** |
| --- | --- | --- | --- | --- | --- | --- | --- |
| ΔTc | 164.5 (42.35) | 54.14 (14.42) | ＜0.001 | 135.6 (68.17) | 79.2 (46.15) | 0.101 | 0.815 (0.592-1.000) |
| ΔTh | 153.6 (48.72) | 100.6 (44.06) | 0.047 | 160.0 (42.38) | 82.2 (22.80) | 0.001 | 0.870 (0.682-1.000) |
| ΔNK | 133.0 (29.6) | 81.3 (39.54) | 0.013 | 129.4 (34.36) | 78.0 (36.13) | 0.015 | 0.843 (0.638-1.000) |

Abbreviations: CR, complete response; PR, partial response; SD, stable disease; PD, progressive disease; M± S, mean ± standard deviation; Tc, cytotoxic T cells; Th, helper T cells; NK cells, natural killer cells; AUC, area under the curve.
